# Supplementary material for: Amplitude mode in the planar triangular antiferromagnet Na0.9MnO2
Source: Nat Commun. 2018 Jun 5;9:2188. doi: 10.1038/s41467-018-04601-1 (PMC5988795; doi:10.1038/s41467-018-04601-1)
Supplement: Supplementary file 1 — Supplementary Information [file 41467_2018_4601_MOESM1_ESM.pdf]

Supplementary Information

Amplitude mode in the planar triangular antiferromagnet $Na_{0.9}MnO_2$

Dally et al.

SUPPLEMENTARY NOTE 1: LINEAR SPIN WAVE THEORY FOR COUPLED ANTIFERROMAGNETIC CHAINS WITH EASY-AXIS ANISOTROPY

For the case of weakly coupled spin chains with single-ion anisotropy, we consider the Hamiltonian

$$H = \frac{1}{2}\sum_{ij} J_{ij}\mathbf{S}_i \cdot \mathbf{S}_j - \sum_i D(S_i^z)^2, \tag{1}$$

with spins described on an anisotropic triangular lattice using the basis vectors $\mathbf{e}_1 = (b, 0)$, $\mathbf{e}_2 = (-\frac{b}{2}, \frac{a}{2})$, and $\mathbf{e}_3 = (-\frac{b}{2}, -\frac{a}{2})$ as illustrated in Supplementary Figure 1. We introduce the standard Holstein-Primakoff representation for the spins. On the spin up sublattice, the state excited by $\hat{n}_i$ quanta is represented as

$$S_i^z = S - \hat{n}_i, \qquad S_i^+ = \sqrt{2S - \hat{n}_i}a_i, \qquad S_i^- = a_i^\dagger\sqrt{2S - \hat{n}_i}, \tag{2}$$

with $\hat{n}_i = a_i^\dagger a_i$. Similarly, for spins on the spin down sublattice

$$S_i^z = -S + \hat{n}_i, \qquad S_i^+ = b_i^\dagger\sqrt{2S - \hat{n}_i}, \qquad S_i^- = \sqrt{2S - \hat{n}_i}b_i, \tag{3}$$

and $\hat{n}_i = b_i^\dagger b_i$. Here $a_i, a_i^\dagger$ and $b_i, b_i^\dagger$ are canonical Bose operators.

Considering only nearest neighbor J_1 and next nearest neighbor J_2 couplings, the Hamiltonian becomes,

$$\begin{aligned} H = \sum_{i\in \mathrm{A}} & J_1(\mathbf{S}_i^{\mathrm{A}} \cdot \mathbf{S}_{i+e_1}^{\mathrm{B}} + \mathbf{S}_i^{\mathrm{A}} \cdot \mathbf{S}_{i-e_1}^{\mathrm{B}}) + J_2(\mathbf{S}_i^{\mathrm{A}} \cdot \mathbf{S}_{i+e_2}^{\mathrm{B}} + \mathbf{S}_i^{\mathrm{A}} \cdot \mathbf{S}_{i-e_2}^{\mathrm{B}}) \\ & + J_2\mathbf{S}_i^{\mathrm{A}} \cdot \mathbf{S}_{i+e_3}^{\mathrm{A}} + J_2\mathbf{S}_{i+e_1}^{\mathrm{B}} \cdot \mathbf{S}_{i+e_1+e_3}^{\mathrm{B}} - D[(S_i^{\mathrm{A},z})^2 + (S_{i+e_1}^{\mathrm{B},z})^2], \end{aligned} \tag{4}$$

where A and B are the spin up and spin down sublattices, respectively. The Holstein-Primakoff transformations defined in Eqs.(2),(3), applied to the spin terms gives

$$(S_i^z)^2 = (S - \hat{n}_i)^2 = S^2 - 2S\hat{n}_i + \hat{n}_i^2 \tag{5a}$$

$$\begin{aligned} \mathbf{S}_i^{\mathrm{A}} \cdot \mathbf{S}_j^{\mathrm{B}} &= (S - \hat{n}_i)(-S + \hat{n}_j) + \frac{1}{2}\sqrt{2S - \hat{n}_i}a_i\sqrt{2S - \hat{n}_j}b_j + \frac{1}{2}a_i^\dagger\sqrt{2S - \hat{n}_i}b_j^\dagger\sqrt{2S - \hat{n}_j} \\ &\approx -S^2 + S(\hat{n}_i + \hat{n}_j) + S(a_ib_j + a_i^\dagger b_j^\dagger) + O(a^4 ...) \end{aligned} \tag{5b}$$

$$\begin{aligned} \mathbf{S}_i^{\mathrm{A}} \cdot \mathbf{S}_j^{\mathrm{A}} &= (S - \hat{n}_i)(S - \hat{n}_j) + \frac{1}{2}\sqrt{2S - \hat{n}_i}a_ia_j^\dagger\sqrt{2S - \hat{n}_j} + \frac{1}{2}a_i^\dagger\sqrt{2S - \hat{n}_i}\sqrt{2S - \hat{n}_j}a_j \\ &\approx S^2 - S(\hat{n}_i + \hat{n}_j) + S(a_ia_j^\dagger + a_i^\dagger a_j) + O(a^4 ...) \end{aligned} \tag{5c}$$

$$\mathbf{S}_i^{\mathrm{B}} \cdot \mathbf{S}_j^{\mathrm{B}} \approx S^2 - S(\hat{n}_i + \hat{n}_j) + S(b_i^\dagger b_j + b_ib_j^\dagger) + O(b^4 ...). \tag{5d}$$

Grouping terms to second order in ladder operators gives $H = E_0 + H_2$ where E_0 is an offset and

$$\begin{aligned} H_2 = \sum_{i\in \mathrm{A}} & SJ_1[2\hat{n}_i^{\mathrm{A}} + \hat{n}_{i+e_1}^{\mathrm{B}} + \hat{n}_{i-e_1}^{\mathrm{B}} + a_i(b_{i+e_1} + b_{i-e_1}) + h.c.] + J_2S[2\hat{n}_i^{\mathrm{A}} + \hat{n}_{i+e_2}^{\mathrm{B}} + \hat{n}_{i-e_2}^{\mathrm{B}} + a_i(b_{i+e_2} + b_{i-e_2}) + h.c.] \\ & + SJ_2[-\hat{n}_i^{\mathrm{A}} - \hat{n}_{i+e_3}^{\mathrm{A}} - \hat{n}_{i+e_1}^{\mathrm{B}} - \hat{n}_{i+e_1+e_3}^{\mathrm{B}} + a_i^\dagger a_{i+e_3} + b_{i+e_1}^\dagger b_{i+e_1+e_3} + b_{i+e_1+e_3}^\dagger b_{i+e_1}] + 2DS(\hat{n}_i^{\mathrm{A}} + \hat{n}_{i+e_1}^{\mathrm{B}}). \end{aligned} \tag{6}$$

Transforming this H_2 term into momentum space gives

$$H_2/S = \sum_{\mathbf{k}} 2[J_1 + D + J_2\cos(\mathbf{k}\cdot\mathbf{e}_3)][a_{\mathbf{k}}^\dagger a_{\mathbf{k}} + b_{\mathbf{k}}^\dagger b_{\mathbf{k}}] + 2[J_1\cos(\mathbf{k}\cdot\mathbf{e}_1) + J_2\cos(\mathbf{k}\cdot\mathbf{e}_2)][a_{\mathbf{k}}b_{-\mathbf{k}} + a_{\mathbf{k}}^\dagger b_{-\mathbf{k}}^\dagger]. \tag{7}$$

This has the form

$$H_2/S = \sum_{\mathbf{k}}[\omega_{\mathbf{k}}(a_{\mathbf{k}}^\dagger a_{\mathbf{k}} + b_{-\mathbf{k}}^\dagger b_{-\mathbf{k}}) + \lambda_{\mathbf{k}}(a_{\mathbf{k}}b_{-\mathbf{k}} + a_{\mathbf{k}}^\dagger b_{-\mathbf{k}}^\dagger)], \tag{8}$$

with $\omega_{\mathbf{k}} = 2[J_1 + D + J_2\cos(\mathbf{k}\cdot\mathbf{e}_3)]$ and $\lambda_{\mathbf{k}} = 2[J_1\cos(\mathbf{k}\cdot\mathbf{e}_1) + J_2\cos(\mathbf{k}\cdot\mathbf{e}_2)]$. This can be simplified by applying the Bogoliubov transformation,

$$a_{\mathbf{k}} = \cosh\theta_{\mathbf{k}}\alpha_{\mathbf{k}} + \sinh\theta_{\mathbf{k}}\beta^{\dagger}_{-\mathbf{k}}, \qquad b_{\mathbf{k}} = \cosh\theta_{\mathbf{k}}\beta_{-\mathbf{k}} + \sinh\theta_{\mathbf{k}}\alpha^{\dagger}_{\mathbf{k}}. \tag{9}$$

The parameter $\theta_{\mathbf{k}}$ is determined by inserting Eq. (9) into Eq. (8) and requiring that the

$$H_2/S = \sum_{\mathbf{k}} \frac{\Omega_{\mathbf{k}}}{S}(\alpha^{\dagger}_{\mathbf{k}}\alpha_{\mathbf{k}} + \beta^{\dagger}_{-\mathbf{k}}\beta_{-\mathbf{k}}) \tag{10}$$

Using the general expression, we obtain two conditions:

$$\omega_{\mathbf{k}}\sinh 2\theta_{\mathbf{k}} + \lambda_{\mathbf{k}}\cosh 2\theta_{\mathbf{k}} = 0, \tag{11}$$

$$\frac{\Omega_{\mathbf{k}}}{S} = \omega_{\mathbf{k}}\cosh 2\theta_{\mathbf{k}} + \lambda_{\mathbf{k}}\sinh 2\theta_{\mathbf{k}}. \tag{12}$$

The solution to Eq. (11) is

$$\sinh 2\theta_{\mathbf{k}} = \frac{-\lambda_{\mathbf{k}}}{\sqrt{\omega_{\mathbf{k}}^2 - \lambda_{\mathbf{k}}^2}} \quad , \quad \cosh 2\theta_{\mathbf{k}} = \frac{\omega_{\mathbf{k}}}{\sqrt{\omega_{\mathbf{k}}^2 - \lambda_{\mathbf{k}}^2}}, \tag{13}$$

assuming $\omega_{\mathbf{k}} > 0$, which is satisfied here. This gives the single magnon spin energy

$$\Omega/S = \omega_{\mathbf{k}}\cosh 2\theta_{\mathbf{k}} + \lambda_{\mathbf{k}}\sinh 2\theta_{\mathbf{k}} = \sqrt{\omega_{\mathbf{k}}^2 - \lambda_{\mathbf{k}}^2}. \tag{14}$$

The expression in the text is obtained by using the explicit forms for $\omega_{\mathbf{k}}$ and $\lambda_{\mathbf{k}}$ given above, and rewriting them in the reciprocal lattice units defined in the main text: $\mathbf{k}\cdot\mathbf{e}_1 = 2\pi K$, $\mathbf{k}\cdot\mathbf{e}_2 = \pi(H-K)$, and $\mathbf{k}\cdot\mathbf{e}_3 = -\pi(H+K)$.

SUPPLEMENTARY NOTE 2: TWO MAGNON BOUND STATE IN ANTIFERROMAGNETIC CHAIN WITH EASY-AXIS ANISOTROPY

Spin wave theory and path integral

In this section, we expand about the 0 K antiferromagnetic ground state and ignore the coupling J_2 between chains for simplicity, since it is very small and does not signficantly influence the magnon binding in this limit. This reduces the problem to a single Heisenberg chain with single-ion Ising anisotropy:

$$H = \sum_n \left[J\mathbf{S}_n\cdot\mathbf{S}_{n+1} - D\left(S_n^z\right)^2\right], \tag{15}$$

with $J = J_1, D > 0$, and we assume $D \ll J$. As in the prior subsection, we apply spin wave theory using the Holstein-Primakoff relations in Eq. (2), (3). In the departure from the prervious subsection, we, however, do not, at this point, truncate to quadratic order in the boson operators. The Holstein-Primakoff representation inserted into H gives a complicated expression for $H[a^{\dagger}, a, b^{\dagger}, b]$, which we do not write explicitly. Now we write the path integral representation of this boson problem, which has the action

$$S = \int d\tau \left\{\sum_n \left(\bar{a}_{2n}\partial_\tau a_{2n} + \bar{b}_{2n+1}\partial_\tau b_{2n+1}\right) + H[\bar{a}, a, \bar{b}, b]\right\} \tag{16}$$

It is very convenient to make the transformation $b \leftrightarrow \bar{b}$. This leads to

$$S \to \int d\tau \left\{\sum_n \left(\bar{a}_{2n}\partial_\tau a_{2n} - \bar{b}_{2n+1}\partial_\tau b_{2n+1}\right) + H[\bar{a}, a, b, \bar{b}]\right\} \tag{17}$$

The benefit of this form is that the U(1) symmetry of S^z conservation is manifest, and the resulting action has no anomalous terms. It additionally is convenient for a field-theoretical analysis, which allows a simplified treatment of the *low energy* physics at small momentum and low energy. This is advantageous because going beyond quadratic order otherwise introduces a very algebraically complicated problem.

Continuum field theory

Now we proceed to convert this into a continuum field theory. We first make the transformation

$$a = \frac{1}{\sqrt{2}}(\eta + \phi), \qquad b = \frac{1}{\sqrt{2}}(\eta - \phi), \tag{18}$$

and perform a gradient expansion, i.e. take a continuum limit. We let $x = 2n$ and $\sum_n \to \int dx/(2)$. The action can be expanded to quartic order as $S = S_0 + S_2 + S_4$, with

$$S_2 = \int d\tau dx \left\{ \frac{1}{2}(\bar{\eta}\partial_\tau \phi - \eta \partial_\tau \bar{\phi}) + S(2J + D)\bar{\eta}\eta + \frac{JS}{2}\partial_x \bar{\phi}\partial_x \phi + DS\bar{\phi}\phi \right\}. \tag{19}$$

Here we see that the η field has a large mass of order JS, and hence dropped a second spatial derivative term of η, which is sub-dominant. The contribution of order DS to the mass of η may also be neglected. Now we can integrate out η to obtain an action for ϕ alone:

$$S_2 \to \int d\tau dx \left\{ \frac{\partial_\tau \bar{\phi}\partial_\tau \phi}{8JS} + \frac{JS}{2}\partial_x \bar{\phi}\partial_x \phi + DS\bar{\phi}\phi \right\}. \tag{20}$$

It is convenient to make the following rescalings:

$$\tau \to \frac{\tau}{2JS}, \qquad \phi \to 2\phi. \tag{21}$$

The transformation on τ is equivalent to measuring energy in units of $2JS$. This brings the quadratic action into canonical form:

$$S_2 \to \int d\tau dx \left\{ \partial_\tau \bar{\phi}\partial_\tau \phi + \partial_x \bar{\phi}\partial_x \phi + m^2 \bar{\phi}\phi \right\}, \tag{22}$$

with $m^2 = 2D/J$. With these rescalings, the Green's function is just

$$\langle \phi\bar{\phi} \rangle = G(k, \omega_n) = \frac{1}{\omega_n^2 + k^2 + m^2}. \tag{23}$$

Now, carrying through all the same rescalings, the quartic part of the action reads

$$S_4 \to \frac{1}{S}\int d\tau dx \left\{ -m^2(\bar{\phi}\phi)^2 + \frac{1}{2}\left[\bar{\phi}^2(\partial_x \phi)^2 + (\partial_x \bar{\phi})^2 \phi^2 \right] \right\}. \tag{24}$$

Here we set the massive field $\eta \to 0$, and kept only leading terms in gradients, using the dimension counting $m \sim \partial_x$.

Diagrammatic analysis

We first consider the longitudinal spin fluctuations and magnon bound state in a diagrammatic analysis. The correlation function of the longitudinal spin fluctuations,

$$C(x, \tau) = \left\langle (\bar{\phi}\phi)_{x,\tau}(\bar{\phi}\phi)_{0,0} \right\rangle, \tag{25}$$

is the leading term in the fluctuations of the spin along the z axis, c.f. Eq. (2). Its Fourier transform is diagrammatically

$C(k, i\omega_n) =$ 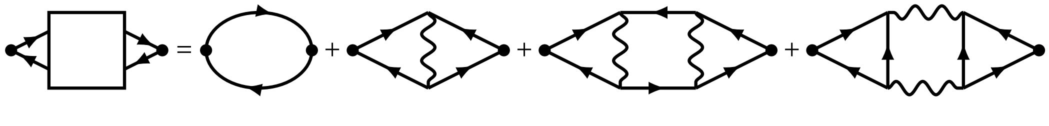 $+ \cdots .$

(26)

If we neglect the final diagram in Eq. (26), we see the first three terms in a ladder series. We will sum this series to obtain what is hopefully the leading approximation of longitudinal correlation function. From Eq. (24) we can read off the vertex

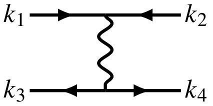

$$= \frac{1}{S}\left[-m^2 - \frac{1}{2}\left(k_1 k_2 + k_3 k_4\right)\right]. \tag{27}$$

Note that the entire vertex is of order $1/S$, as expected from the semi-classical nature of the spin wave expansion. It consists of two terms. The first is parametrically small in the anisotropy-induced mass, while the second represents magnon interactions that would be present in the isotropic Heisenberg limit. The latter interactions have larger amplitude but vanish in the small momentum limit.

Next consider the special case of zero momentum, i.e. at the Bragg peak position, $k = 0$. In this case a representative term in the ladder sum has internal momenta as indicated here:

$$C(k = 0, i\omega_n) = \cdots + \tag{28}$$

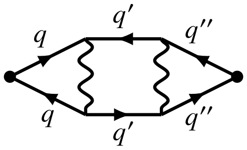

$$+ \cdots$$

Here we have not shown the internal frequency variables explicitly, to keep the diagram uncluttered. Comparing with the vertex in Eq. (27), one can can see that ladder diagrams with this structure enjoy an important simplification. The momentum integration over q corresponding to the left-most loop in Eq. (28) contains a denominator (the product of two Green's functions) which is even in q, but the momentum-dependent vertex factors from the second term in Eq. (27) is *odd* in q, and thus vanish under integration. This holds true by induction, working from left to right in the diagram, for every vertex. Therefore, within the ladder sum, we may replace the vertex in Eq. (27) by the constant term $-m^2/S$. One obtains then the usual RPA type form for the ladder sum,

$$C(k = 0, i\omega_n) = \frac{\Pi(0, i\omega_n)}{1 - 4\frac{m^2}{S}\Pi(0, i\omega_n)}, \tag{29}$$

with the polarization bubble

$$\Pi(0, i\omega_n) = \int \frac{dk d\Omega}{(2\pi)^2} G(k, \Omega) G(k, \Omega + \omega_n) \tag{30}$$

Performing the Ω integral and simplifying, we obtain

$$\Pi(0, i\omega_n) = \int \frac{dk}{2\pi} \frac{1}{\epsilon(k)} \frac{1}{\omega_n^2 + (2\epsilon(k))^2}, \tag{31}$$

with $\epsilon(k) = \sqrt{k^2 + m^2}$. One can see from Eq. (31) that the polarization bubble is analytic and real for $|\omega| < 2m$, i.e. below the threshold for 2-magnon creation. We are seeking a bound state, so are interested in this regime. The integral is fully convergent at large k, so we can take the limits to be infinite, and rescale $k \longrightarrow mk$ and take $i\omega_n \longrightarrow \omega$ to obtain

$$\Pi(0, \omega) = \frac{1}{m^2} \int \frac{dk}{2\pi} \frac{1}{\sqrt{k^2 + 1}} \frac{1}{-(\omega/m)^2 + 4(k^2 + 1)}. \tag{32}$$

The condition for a bound state is the vanishing denominator of the RPA expression in Eq. (29), i.e.

$$\frac{4m^2}{S}\Pi(0, \omega) = 1. \tag{33}$$

We see that for large S (which we are assuming), the polarization must be large to achieve this condition. This implies the bound state occurs close to the edge of the continuum, i.e. $2m - \omega \ll m$, so that the integral in Eq. (32) is large. Let

$$\omega = 2m(1 - \delta), \tag{34}$$

with $\delta \ll 1$. Then the integral in Eq. (32) is dominated by momenta of order δ, so we can neglect the factor of k^2 in the square root in the denominator of the first term in the integrand. Then the integral can be carried out and one obtains the solution for δ,

$$\delta \approx \frac{1}{8S^2}. \tag{35}$$

Note that from Eq. (34), δ is defined as the binding energy divided by the two-magnon gap. So if we define the one-magnon gap Δ, then we have

$$\frac{E_{\text{binding}}}{\Delta} = \frac{1}{4S^2}. \tag{36}$$

Quantum mechanics approach

Now the simplicity of the result, and especially the dropping out of the complicated vertex factors in the ladder diagrams, suggests there is a simpler explanation. Intuitively, the binding energy is small even compared to the small gap m (by the $1/S^2$ factor). This suggests the bound state is composed of wavevectors k_b which are small compared to the mass m. In that case, a non-relativistic approximation holds. The condition $k_b \ll m$ would justify the neglect of the gradient terms in the interaction, Eq. (24). Then one ought to reduce the problem to one of just two particles interaction via a delta function potential of strength $1/S$.

To see how this works in practice, we introduce the mode expansion for a complex scalar field, standard in field theory textbooks (or from the analysis of acoustic phonons):

$$\phi(x) = \frac{1}{\sqrt{L}} \sum_k \frac{1}{\sqrt{2\epsilon_k}} \left(a_k e^{ikx} + b_k^\dagger e^{-ikx} \right), \tag{37}$$

where the a_k, b_k are two independent sets of canonical fermion annihilation operators. They represent magnons with spin $S^z = +1$ and $S^z = -1$, respectively. The quadratic Hamiltonian corresponding to S_2 is then

$$H_2 = \sum_k \epsilon_k \left(a_k^\dagger a_k + b_k^\dagger b_k \right). \tag{38}$$

The longitudinal bound state should be considered a state with one of each type of fermion. Hence we insert the mode expansion into the Hamiltonian corresponding to S_4 (which is just the same as S_4 without the τ integral), and keep only terms which describe a-b interactions:

$$\begin{aligned} H_4 &\to \frac{1}{S}\frac{1}{L} \sum_{k_1\cdots k_4} \frac{1}{\sqrt{\epsilon_1\epsilon_2\epsilon_3\epsilon_4}} \left[-m^2 + \frac{k_1k_2 + k_3k_4}{2} \right] a_{k_1}^\dagger b_{k_2} b_{k_3}^\dagger a_{k_4} \delta_{k_1+k_3,k_2+k_4} \\ &= \frac{1}{S}\frac{1}{L} \sum_{k,k',q} \frac{1}{\sqrt{\epsilon_{k+q}\epsilon_{k'+q}\epsilon_k\epsilon_{k'}}} \left[-m^2 + \frac{(k+q)(k'+q) + kk'}{2} \right] a_{k+q}^\dagger a_k b_{k'}^\dagger b_{k'+q}. \end{aligned} \tag{39}$$

If only states with momenta $|k| \ll m$ are involved, the second term in the square brackets above is negligible, and moreover we may approximate $\epsilon_k \approx m$. Then we have

$$H_4 \approx -\frac{1}{S}\frac{1}{L} \sum_{k,k',q} a_{k+q}^\dagger a_k b_{k'}^\dagger b_{k'+q} = -\frac{1}{S} \int dx\, a^\dagger(x) a(a)\, b^\dagger(x) b(x). \tag{40}$$

We recognize an attractive delta-function interaction between a and b particles, with strength $u = -1/S \ll 1$. In the same limit $|k| \ll m$, we may approximate $\epsilon_k \approx m + k^2/2m$ in H_2 in Eq. (38). The problem is now a fully non-relativistic one. For the case of one a and one b particle, we can write the first quantized Hamiltonian

$$\mathcal{H} = 2m - \frac{\partial_{x_1}^2}{2m} - \frac{\partial_{x_2}^2}{2m} - u\,\delta(x_1 - x_2). \tag{41}$$

The ground state of this Hamiltonian has zero total momentum and the wavefunction

$$\Psi(x_1, x_2) = \frac{\lambda}{2} \mathrm{e}^{-\lambda|x_1 - x_2|}, \tag{42}$$

with $\lambda = mu/2$ and the energy $E = 2m - E_\mathrm{b}$, with binding energy $E_\mathrm{b} = mu^2/4$. Using this result and $u = 1/S$, and writing, in these units, the single magnon gap $\Delta = m$, we obtain

$$\frac{E_\mathrm{b}}{\Delta} = \frac{1}{4S^2}. \tag{43}$$

This is the same as we found in the diagrammatic treatment.

What have we gained? Well we don't need the diagrams! But we also gain a bit more understanding. We see that that $\lambda/m = u/2 = 1/2S \ll 1$, which justifies the assumption $k \ll m$. Furthermore, due to the Galilean invariance of Eq. (41), the general solution for the ground state of Eq. (41) with non-zero momentum k is just that of a particle with mass $2m$, i.e.

$$E(k) = 2m - \frac{mu^2}{4} + \frac{k^2}{4m}. \tag{44}$$

So the bound state has a higher mass than the single spin wave, and appears consequently to have a flatter dispersion. This is, however, only valid for $k \ll m$. More generally, the dispersion for larger k will appear more relativistic.

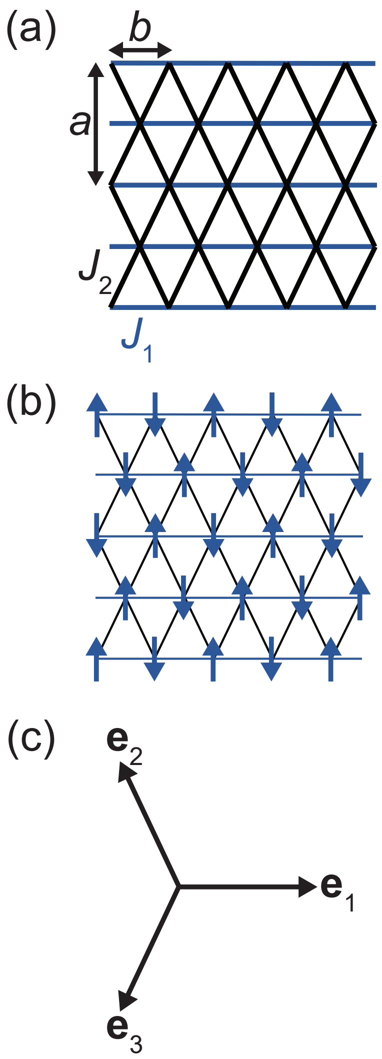

Supplementary Figure 1 | Illustration of the spin lattice in $NaMnO_2$. (a) The two-dimensional Mn lattice in the *ab*-plane with nearest neighbor exchange coupling, J_1, shown as blue lines, and next-nearest neighbor exchange coupling, J_2, shown as black lines. (b) The long-range magnetic order, $\mathbf{k} = (\frac{1}{2}, \frac{1}{2}, 0)$, of the Mn spins (blue arrows) projected onto the *ab*-plane. (c) Vector definitions as used in the spin wave calculation.

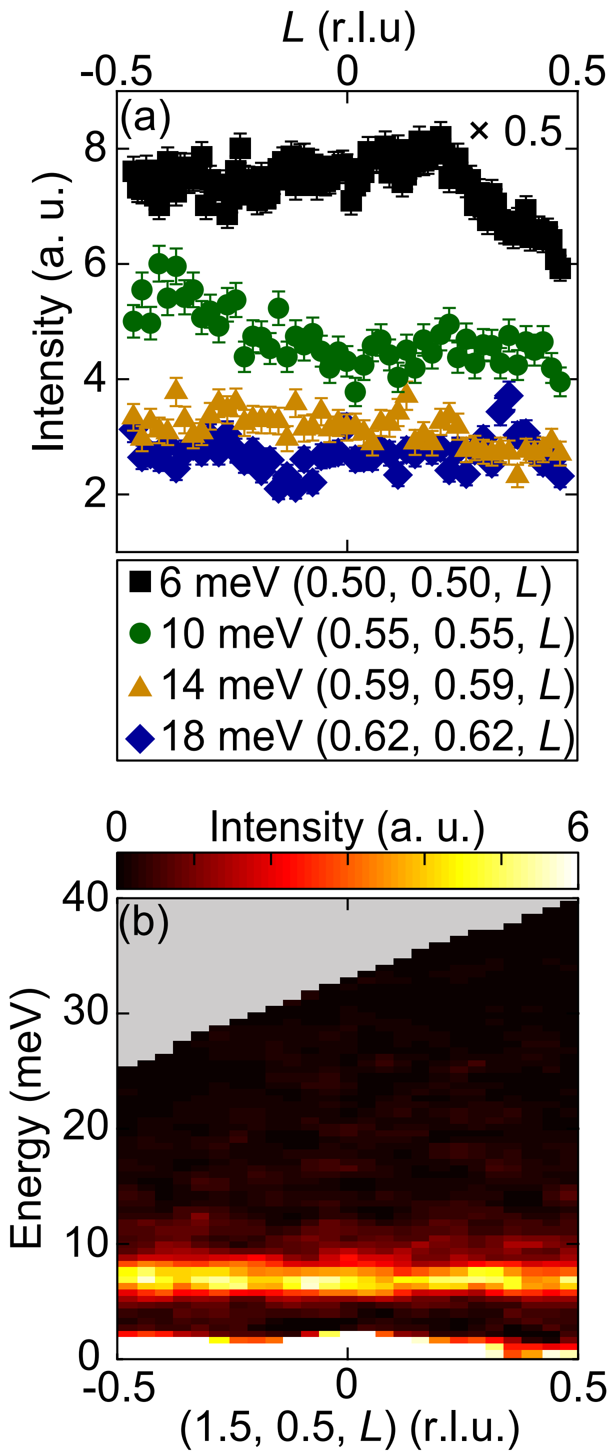

Supplementary Figure 2 | Dispersion of spin waves along the *L*-axis. (a) Momentum scans collected via triple-axis measurements at select energies at 2.5 K. Error bars represent one standard deviation. (b) *L* dependence of the zone center magnons measured via time-of-flight measurements at 4 K. Data has been integrated about the (1.5, 0.5, 0) magnetic zone center with widths *H*=[1.48, 1.52] and *K*=[0.48, 0.52].

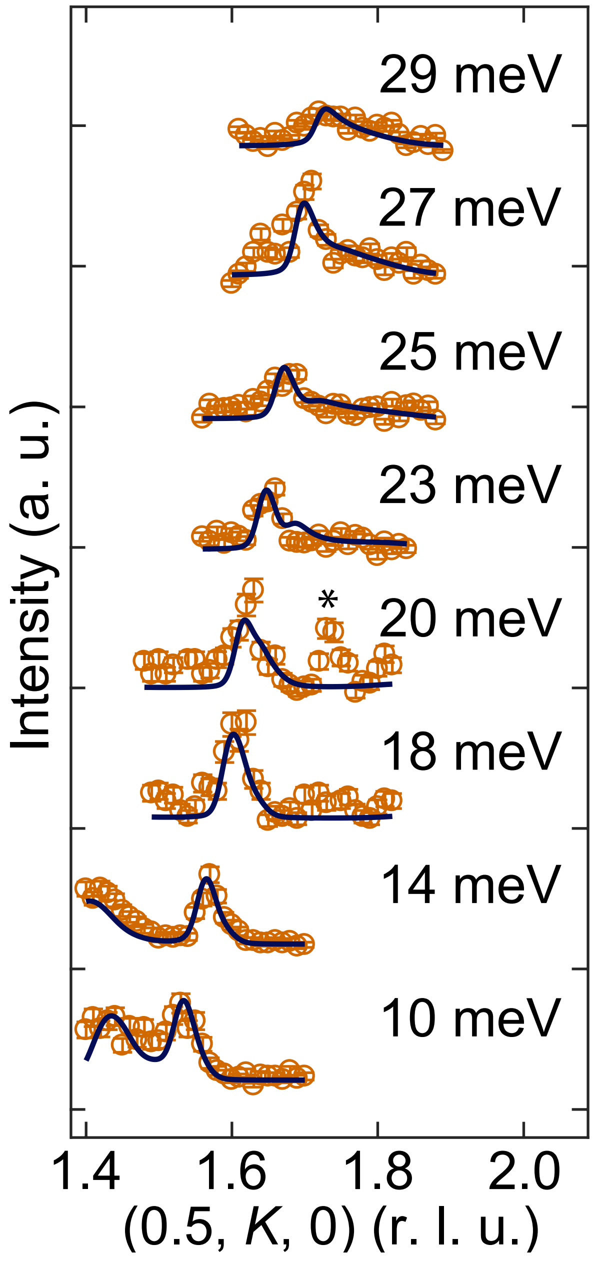

Supplementary Figure 3 | Higher energy triple-axis data collected about the (0.5, 1.5, 0) zone center at 4 K. Data show momentum scans along the K-axis at various energies. Data at different energies are offset for clarity. Solid lines denote fits to the dispersion using the multidomain J_1-J_2-D model described in the main text. The star above the 20 meV data denotes a spurious background feature. Error bars represent one standard deviation.

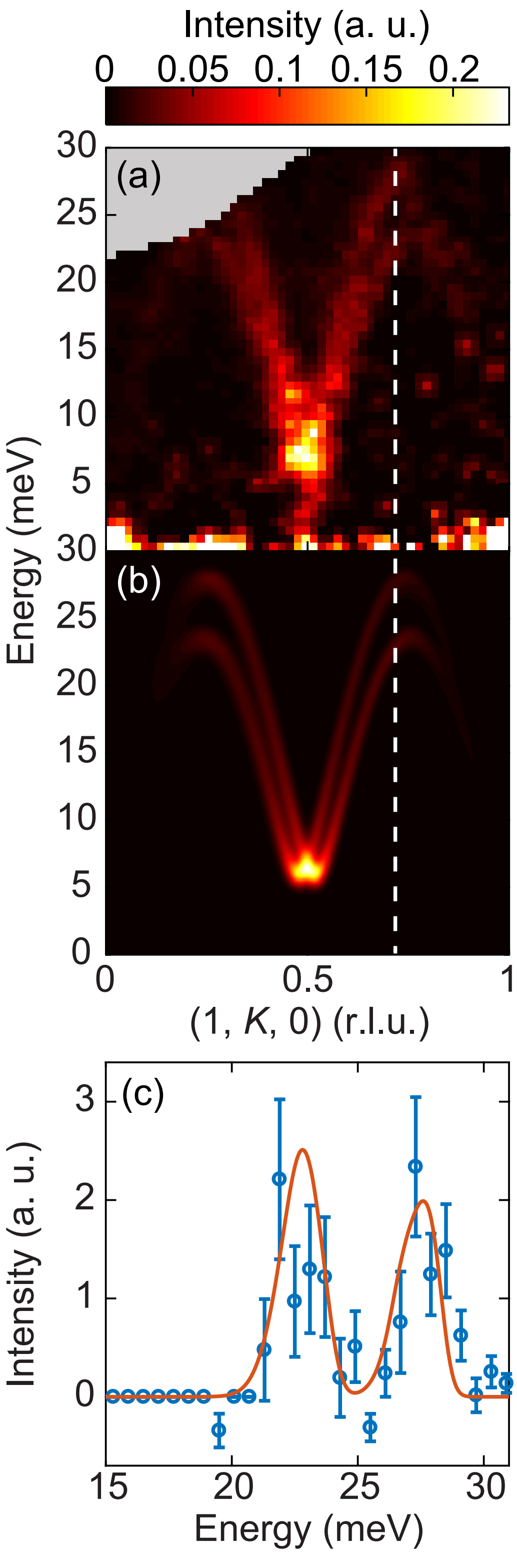

Supplementary Figure 4 | Spin wave spectra collected at 4 K via time-of-flight measurements compared with the J_1-J_2-D multidomain model described in the main text. Panels (a), (b), and (c) show the map of scattering data about the zone center (1, 0.5, 0), modeled intensities, and a representative cut through the data and model respectively. The vertical dashed line denotes the position of the constant momentum cut through both the data and the multidomain model. In panels (a) and (b) intensities were integrated out of the plane through $0.9<H<1.1$ and $-0.1<L<0.1$. The cut in panel (c) was further integrated through $0.70<K<0.74$ The error bars in (c) represent one standard deviation.
